# Assessing and Managing the Metabolic Syndrome in Children and Adolescents

**DOI:** 10.3390/nu11081788

**Published:** 2019-08-02

**Authors:** Mark D. DeBoer

**Affiliations:** Department of Pediatrics, University of Virginia, Charlottesville, VA 22908, USA; deboer@virginia.edu; Tel.: +1-434-924-9833; Fax: +1-434-924-9181

**Keywords:** metabolic syndrome, obesity, insulin resistance, risk, pediatric, adolescent

## Abstract

The metabolic syndrome (MetS) is a group of cardiovascular risk factors that are associated with insulin resistance and are driven by underlying factors, including visceral obesity, systemic inflammation, and cellular dysfunction. These risks increasingly begin in childhood and adolescence and are associated with a high likelihood of future chronic disease in adulthood. Efforts should be made at both recognition of this metabolic risk, screening for potential associated Type 2 diabetes, and targeting affected individuals for appropriate treatment with an emphasis on lifestyle modification. Effective interventions have been linked to reductions in MetS—and in adults, reductions in the severity of MetS have been linked to reduced diabetes and cardiovascular disease.

## 1. Introduction

The roots of cardiovascular disease—the most common cause of mortality among adults worldwide—begin in childhood [1], underscoring the need to identify and intervene in at-risk children [2]. These issues have become even more important in light of the global obesity epidemic, in which over 100 million children worldwide are obese [3], including in developing areas more commonly associated with food scarcity [4]. One predictor of future risk is the metabolic syndrome (MetS), a cluster of cardiovascular risk factors including central obesity (typically measured by high waist circumference or high BMI), hypertension, high fasting triglycerides, low high density lipoprotein (HDL) cholesterol and high fasting glucose [5]. These individual components of MetS occur together more often than would be expected by chance—as though they are driven by similar underlying processes that lead to insulin resistance, including cellular dysfunction in adipocytes, myocytes, and hepatocytes; oxidative stress; and cellular inflammation [6,7]. In addition to predicting cardiovascular disease (CVD), MetS is also a predictor of future Type 2 diabetes among children [8,9].

This review addresses means of assessing MetS in children and adolescents, the implications of altered metabolic status, and approaches toward intervening among affected children and adolescents.

## 2. What is MetS?

MetS, at its core, appears to be due to dysregulated cellular metabolism [7], leading to insulin resistance. A central driver appears to be an excess of central obesity, with visceral adipocytes releasing chemo-attractants, contributing to infiltration by macrophages and release of cytokines and an overall increase in systemic inflammation [6]. Further adipocyte dysfunction includes reduced production of the adipokine adiponectin (which appears to be in the causative pathway of insulin resistance) [10] and higher release of free fatty acids [11]. In peripheral tissues, these high levels of free fatty acids and triglycerides alter mitochondrial function and increase the degree of oxidative stress, with an overall effect of reductions in insulin’s ability to stimulate glucose transporters to the cell surface [7]. The degree of insulin resistance results in heightened need for insulin production, and glucose levels rise as the resistance exceeds the ability of the pancreatic beta cells to release adequate amounts of insulin, ultimately contributing to risk for Type 2 diabetes [12]. Further downstream effects include hypertension and reduced levels of HDL cholesterol, both of which contribute additional risk to cardiovascular disease [2]. This multifaceted process has made it difficult to adequately target—though, as we will see, weight reduction to decrease the central adiposity and exercise to increase energy utilization have been effective in reducing the metabolic abnormalities.

## 3. Clinical Measures of MetS

### 3.1. Evaluation among Adults

The first observations regarding MetS were related to linking distinct abnormalities in the individual components [5], and this approach ultimately led to forming diagnostic criteria that identified individuals with several of these metabolic abnormalities. These criteria were first set for classifying MetS among adults, with the most commonly-used criteria being those of the National Cholesterol Education Program’s Adult Treatment Panel III (ATP-III) [13]. Using ATP-III criteria, an individual is categorized as having MetS if they have measured values that are outside the adult normal range for at least three of the individual MetS components (WC, BP, triglycerides, HDL, glucose—with current diabetes qualifying as an abnormal glucose level even in the absence of an elevated value). Other organizations have proposed slightly different criteria; the World Health Organization criteria utilized results from oral glucose tolerance tests [14], while the International Diabetes Federation (IDF) initially required the presence of central obesity for MetS classification (regardless of how many other MetS abnormalities were present) [15]. The IDF criteria (later harmonized to be in line with ATP-III criteria [16]) also allow for use of separate cut-offs for elevated waist circumference by race/ethnicity, based on evidence demonstrating risk in a specific group [15]. 

Each of these sets of categories above diagnoses MetS on a dichotomous basis (i.e., you either have it or you do not). Among adults, there have been scoring systems that take into account that MetS abnormalities exist on a spectrum. Approaches to this have often consisted of a summation of standardized *z*-scores for each individual component among a defined population of interest. We formulated a score of MetS severity using confirmatory factor analysis that allowed for a weighted contribution of the individual criteria, with these weights varying by sex and racial/ethnicity based on how these components correlated together in each sex and racial/ethnic subgroup [17]. Because this was done using nationally-representative data, these MetS-*z* scores can be used to assess risk in other populations without reformulating the scores based on the distribution of abnormalities for the new population. As compared to dichotomous criteria, use of continuous scores such as this can provide improved power for statistical assessment of MetS-related risk [18]. In addition, whereas dichotomous criteria can only be used to follow for the presence or absence of MetS over time [19], continuous scores are also useful to follow for the risks associated with changes in MetS severity over time [20,21] and how an individual responds to intervention [22]. 

### 3.2. Evaluation among Children

Whereas assessment of MetS in adults relied on criteria established by national or international agencies, assessment among children and adolescents has not been as clear [23]. Most assessments have relied on adaptations that were based on adult criteria, with cut-off values for the individual components that were altered to reflect the more moderate values for these risk factors among adolescents (Table 1) [24,25,26]. The IDF proposed a set of criteria for children that was based on the adult IDF criteria, again requiring abnormal waist circumference for MetS classification [27]. Other criteria have acknowledged the gradual shift over the course of adolescence in normal ranges of the individual components, with cut-offs that change with time [28].

As with the assessment of MetS-related risk in adults, continuous scores have frequently been used in pediatrics. There have been multiple approaches to this, again with most consisting of summation of *z*-scores for a particular underlying population [18]. We again used confirmatory factor analysis in a nationally-representative group of US adolescents age 12–19 to produce MetS severity scores that are weighted to how MetS was manifest by sex and racial/ethnic subgroup [29]. These scores appear to reflect the underlying metabolic disarray in correlating closely with markers of the processes underlying MetS, including C-reactive protein (CRP), uric acid, adiponectin, and insulin [29,30,31,32]. 

Continuous scores are also able to overcome a drawback to sets of criteria, namely, their apparent lack of durability, with a high occurrence of adolescents who toggle between having a diagnosis of MetS or not based on having individual components that are just above or below the cut-off [33,34]. The clinical use of these criteria would likely benefit from incorporation into the electronic medical record [35] but at this time remains less certain, and some researchers have lobbied for an approach that simply targets individual component risk factors [36].

## 4. Epidemiology

The underlying prevalence of MetS in adolescents depends on the set of MetS criteria used, with overall ranges in the US from 1.2%–9.8% using modified ATP-III [31,37] criteria to 4.5%–8.4% using the IDF adolescent criteria [38,39]. Assessments among school-aged children and early adolescents is lower (0.2%–1.2%) [37,40], which is likely because of the strong effects of puberty on insulin resistance. For example, insulin resistance as estimated by measures such as the homeostasis model of insulin resistance—which usually tracks closely with MetS [41]—at age 8 years is half that seen among those at 15 years, consistent with the concept that puberty itself may be involved with the progression of abnormal metabolic processes [42]. 

**Figure 1:**
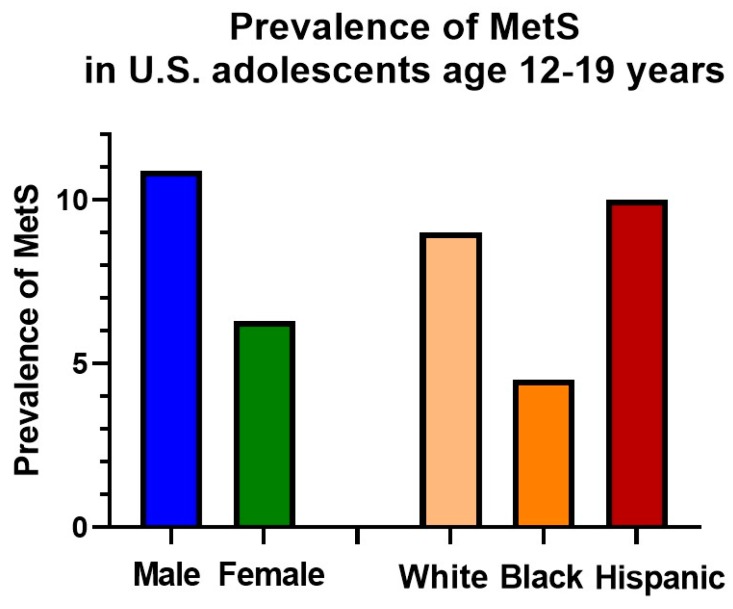
Prevalence of metabolic syndrome in adolescents by sex and race ethnicity. Data are for adolescent participants age 12–19 years from the National Health and Nutrition Examination Survey 1999–2012 as reported in Lee et al. [31].

In addition to variation by age, the prevalence of MetS also varies significantly by sex, with male adolescents having a greater prevalence than females (Figure 1). Interestingly, there is also variation by race/ethnicity, being more common in whites and Hispanics compared to African Americans (Figure 1). This is surprising, given tight associations of MetS with insulin resistance, diabetes, and CVD mortality—all of which are more common in African Americans [38]. The reason for this appears to be more favorable lipid levels in African Americans, particularly lower triglyceride levels, which appear to have a lower baseline levels but do increase with worsening insulin resistance [43]. 

**Figure 2:**
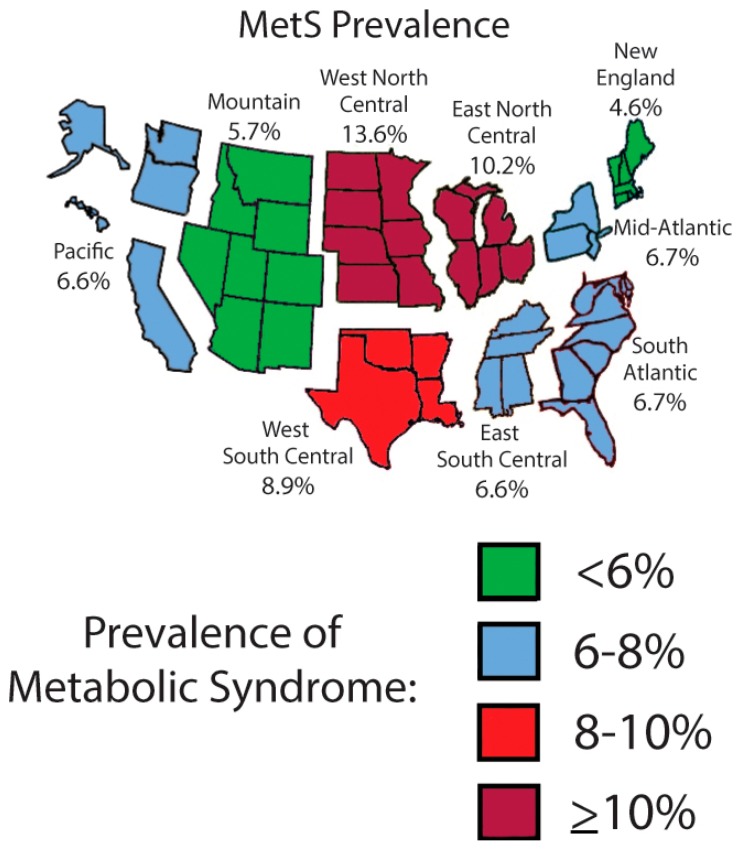
Geographic variation in MetS prevalence among US adolescents. Data are for adolescents age 12–19 years from National Health and Nutrition Examination Survey 1999–2014. (From, DeBoer et al. used with permission.) [45].

Not surprisingly, MetS varies by location, with one meta-analysis estimating a lower prevalence in Europe (2.1%) and the Far East (3.3%) compared to the Americas (4.5%) and the Middle East (6.5%) [44]. Even in the US, prevalence varies by geography, with higher prevalence in the Midwest and South of the US compared to the West and Northeast (Figure 2), with potential implications for allocation of resources by region toward improved lifestyle efforts [45]. While MetS has traditionally been thought of as a problem of developed countries, the increase in pediatric obesity across the globe has made MetS a concern in developing countries as well [46]. The prevalence of MetS in developing areas of the world is likely to worsen with changing diet patterns as calorie-dense foods become increasingly available [46]. Worldwide variation in MetS prevalence is compounded by an apparent increase in susceptibility for obesity and MetS by race/ethnicity [47].

The high prevalence of MetS in adolescents coincides with the current obesity levels, and there has not been evidence of a decrease in the prevalence of MetS by classical criteria. However, there has been a recent decrease in the severity of MetS as assessed by a continuous score over time. This is surprising and appears to be due to decreases in triglycerides, potentially from lower consumption of saturated fat [31].

## 5. Long-Term Risks

The importance of considering MetS in ongoing patient care is driven home by the long-term associations between MetS and future disease. While the downstream sequelae of childhood MetS are usually greater than 10 years in development, studies that followed children for MetS-related characteristics in the 1970s and followed up in the 2000s have demonstrated strong links, with childhood MetS (vs. no MetS) carrying an odds ratio of 2.3–11.5 for future T2DM 14–31 years later [9,48], 2.0 for elevated carotid artery media thickness (a subclinical marker of CVD risk) 14–27 years later [48], and 14.6 for CVD 24–31 years later [8]. In utilizing a MetS Z-score in childhood to assess risk of adult disease 24–31 years later, the odds ratio of increased risk for every 1 standard deviation of the score was 2.7 and 9.8 for future T2DM [49] and CVD [50] (respectively). Moreover, the change in score over time was associated with a further increase in disease risk [49,50], suggesting potential utility in following an adolescent’s MetS score during lifestyle modification treatment as a means of following ongoing risks and motivating further improvements.

It is notable that the prevalence of MetS in the 1970s was only 3.9% [9], compared to the prevalence of 9.8% today [31]. This underscores an enormous risk for future T2DM and CVD based on prevalence of risk factors in the current generation of US adolescents. In addition, MetS is also linked to other obesity-related disease processes, including non-alcoholic fatty liver disease (NAFLD) [51] and renal function [52], with implications for chronic kidney disease [53].

## 6. An Emphasis on Prevention

It should be noted that the best means of reducing the prevalence of MetS in the future is to prevent the occurrence of obesity among children and adolescents. This includes efforts at encouraging an active lifestyle from a young age and preserving of levels of physical activity among younger children (before the usual decline in activity during adolescence [54]). It also includes encouraging families to maintain consumption of fresh foods and avoid energy-dense foods, including as these are increasingly introduced to developed parts of the world [46]. As discussed below, after the development of overweight or obesity, it is difficult to lose excess weight, and stronger efforts should be made at preventing obesity and MetS, including from the standpoint of public policy, including availability of safe spaces for physical activity and healthy nutrition choices in schools [55]. 

## 7. Use of Criteria in Clinical Settings

### Identify Individuals at Highest Need for Assessment

Given the potential long-term sequelae of MetS, the chief roles of MetS as a concept in pediatric clinical care is in-risk identification and patient motivation. However, while the sets of criteria described above provide a means of categorizing MetS and assessing related factors and long-term risk, there is a sense that these criteria are not commonly used in clinical settings [36]. This may be because of the potentially time-intensive nature of comparing measured values of each component with cut-off values. Clearly, the process would be improved by automatic assessments performed by electronic health record systems [35]—though such tools are not commonly available for assessment of pediatric and adolescent MetS. In all settings, including in developing areas in the world, attention should be given to obtaining accurate height and weight measures and assessing how a child’s BMI compares to standardized percentiles for age, such as those of the World Health Organization [56].

Children and adolescents who present with obesity and/or findings associated with MetS should receive extra attention toward reducing long-term risks for future chronic disease. Many of the interventions against MetS (described below) are likely to also have benefits among all overweight children [57]; nevertheless, children classified as having MetS are likely to benefit from additional time and encouragement because of their additional CVD risk factors [36]. In the absence of formal screening for MetS itself (which requires a fasting blood draw and waist circumference measurement), other indicators of insulin resistance and long-term risk for chronic disease may offer alternative means of identifying patients at higher risk. This includes the presence of a strong family history of T2DM [58] or CVD [59] upon questioning or of acanthosis nigricans [60] or hypertension [36] upon physical exam. The presence of these factors in an overweight patient should prompt assessment for potential concurrent Type 2 diabetes, through assessment of symptoms such as polyuria, polydipsia, and unintended weight loss, and through testing HbA1c, with a HbA1c level of 5.7%–6.4% reflective of a pre-diabetes state and a level ≥6.5% consistent with Type 2 diabetes [61].

Discussion regarding how MetS influences a child’s or adolescent’s risk and how reductions in MetS severity may improve their chances of disease development [22] may assist in motivation toward change [2]. Use of such risk identification as a motivator is a key consideration, as the difficulty in care for adolescents with MetS-related risk for future T2D and CVD is not usually in the identification of at-risk adolescents—using either accepted algorithms or looking at higher-risk groups based on epidemiology—or even in advising interventions but in achieving adherence to these interventions, discussed in the following section. 

## 8. Intervention

Because of the strong connection between MetS and obesity, most interventions for MetS have paralleled those for pediatric obesity in general, namely interventions aimed at altering unhealthy lifestyle factors that likely contributed to the metabolic problems in the first place. This includes diets that are high in saturated fat and carbohydrates (and ultimately an excess of overall calories) [31] and physical activity levels that fall far short of recommendations [54]. In addition, further abnormalities in the components of MetS should be addressed if present. In some cases, this could include, for example, treating hypertension with medication. However, because the predominant “lesion” in MetS is the central obesity, the majority of approaches have focused on lifestyle approaches, as addressed in the following section. 

Interventions that have been assessed for efficacy in reducing the proportion of children with MetS have thus focused on altering dietary choices, increasing physical activity, and a combination of both. The goal of these is to thus decrease the ratio of energy ingested vs. energy expended, primarily to reduce the degree of central obesity that drives the metabolic abnormalities. Unfortunately, the unfavorable balance of energy ingested vs. expended has occurred because many children and adolescents have developed suboptimal lifestyle practices due to ease, availability or palatability, and it can be difficult to motivate pediatric patients (and adults as well) to overcome the draw toward these unhealthy lifestyle choices. Effective approaches have included efforts at exploring the motivation of adolescents using techniques such as motivational interviewing, which involves assessments of an individual patient’s readiness to change [62]. These approaches are thus tailored to the individual patient and require the time to probe the patient’s current food choices and level of physical activity—and a degree of flexibility in working out a treatment plan to which the child/adolescent is willing to commit. This kind of approach is able to increase the adherence rate of adolescents to a treatment plan [62].

### 8.1. Dietary Changes

The main approach for dietary changes for children and adolescents as recommended by the American Academy of Pediatrics, the American Heart Association, and the World Health Organization has been an increase in vegetable and fruit consumption and a reduced intake of saturated fat in lieu of unsaturated fat (e.g., olive oil and other vegetable oils), as well as a reduction in sugar intake [56]. A meta-analysis of studies that recommended these changes (though usually as part of an approach combined with changes in physical activity, as compared to no change) demonstrated decreases in BMI. One helpful way of achieving these changes has been through implementation of the Mediterranean diet, which incorporates vegetables and olive oil. With respect to MetS itself, a 16-week trial of the Mediterranean diet among children and adolescents revealed a decrease in MetS prevalence (16% to 5%) among those on the Mediterranean diet compared to no change or worsening in the control group (Velazquez-Lopez). Other studies have supported additional concepts such as that intake of highly-processed food was associated with a 2.5-fold increased risk of MetS [63], and intake of sugar-sweetened beverages (vs. not) is associated with >5-fold risk of MetS [64].While intervention studies assessing effects of eliminating sugar-sweetened beverages on MetS have not been performed, randomized trials have shown some efficacy of sugar-sweetened beverage elimination on improved weight status [55,65], which clearly contributes to risk of MetS. Overall, among patients with MetS, efforts should be made toward reducing consumption of sugar-sweetened beverages, saturated fat, and calorie-dense food (e.g., fast food) and toward increasing consumption of oils and vegetables—likely through negotiating individual changes with adolescents and their families.

### 8.2. Physical Activity Changes

Increases in physical activity serve to maintain or increase total energy expenditure in the face of reduced caloric intake. The US Center for Disease Control and Prevention and the World Health Organization recommend at least 60 minutes of moderate to vigorous physical activity among school-age children and adolescents [66]—though adolescents do particularly poorly in meeting these goals, with <30% engaging in this much activity [54]. As expected, lower levels of physical activity are associated with a greater risk for MetS, including higher levels of a MetS *z*-score [67,68]. Physical activity is particularly good at increasing insulin sensitivity [69].

A goal of increasing physical activity in clinical practice has been through incorporating these activities into a child’s or adolescent’s usual routine. One research group assessed the likelihood of MetS among children and adolescents who rode their bicycle to school (vs. not), finding lower odds of MetS associated with bicycle use [70]. Other approaches have involved providing pedometers to patients and negotiating a daily goal for total steps taken—which the child can document and take personal pride in achieving. Increased walks with family, friends or pets can be a way to ensure continued activity. Finally, participation in sports, either through schools, clubs or regular meetings with friends, can further sustain physical activity and maintain higher energy expenditure. There is a tendency toward declining physical activity with age [54], so encouragement at continuing activity starting at younger ages may be more successful.

### 8.3. Combined Intervention Approaches

The most effective interventions are likely to include a combined approach incorporating reducing calorie intake while increasing energy expenditure. This is because isolated increased physical activity may lead to a compensatory increase in food intake [71], while isolated caloric restriction results in a lowering of basal metabolic rate [72]—while a combination of these approaches aims to prevent these counterproductive reactions. Combined interventions to reduce MetS have focused on nutritional counselling with specific goals for physical activity—usually consisting of at least three weekly exercise sessions [73,74]. This kind of approach can produce dramatic reductions in MetS over time, with one group reporting a decrease from 27% at baseline to 8.3% after one year of combined nutrition and activity interventions [73].

While it is more difficult among children and adolescents to demonstrate the long-term benefits of approaches like this, we evaluated adults in the Diabetes Prevention Program, revealing that the degree of decrease in MetS severity among adults randomized to intensive lifestyle change (compared to those randomized to usual care) was associated with reductions in further odds of developing diabetes or CVD [22]. This underscores the potential utility in following for MetS changes over time during intervention—potentially as a motivator for patients [2] to track changes in future risk. 

Thus, in most clinical intervention care, the medical team makes recommendations for improvements in dietary choices and physical activity concurrently [75] with follow-up to encourage ongoing adherence [76]. Success rates in clinical settings have not always been stellar [77], but children and adolescents who make these changes are likely to improve their metabolic status, with likely long-term health benefits.

## 9. Conclusions

Overall, the high prevalence that we see of MetS currently and the strong associations of MetS with future diabetes and CVD is a great cause of concern that should alarm practitioners who care for children and adolescents. This is particularly true with worsening obesity prevalence worldwide, including in developing areas. Prevention of childhood obesity is critical to reducing future MetS. Screening for obesity and MetS should be incorporated in multiple aspects of pediatric clinical care. Finally, recognizing the presence of MetS and intervening with targeted lifestyle recommendations—with repeated follow-up to encourage adherence—is likely to improve the future health of these children. 

## Figures and Tables

**Table 1:** Pediatric and adolescent metabolic syndrome (MetS) criteria adapted from the National Cholesterol Education Program Adult Treatment Panel III *.

Central Obesity (WC)	High BP (mmHg)	High Triglycerides (mg/dL)	Low HDL (mg/dL)	High Fasting Glucose
WC ≥ 90th percentile [25]	Systolic or diastolic DBP ≥ 90% for age, sex, height [26]	TG ≥ 110 mg/dL (≥1.24 mmol/L)	HDL ≤ 40 mg/dL (<1.03 mmol/L)	≥100 mg/dL (5.6 mmol/L) or known T2DM

* Individuals need to have at least three abnormalities in MetS components to be classified as having MetS.
